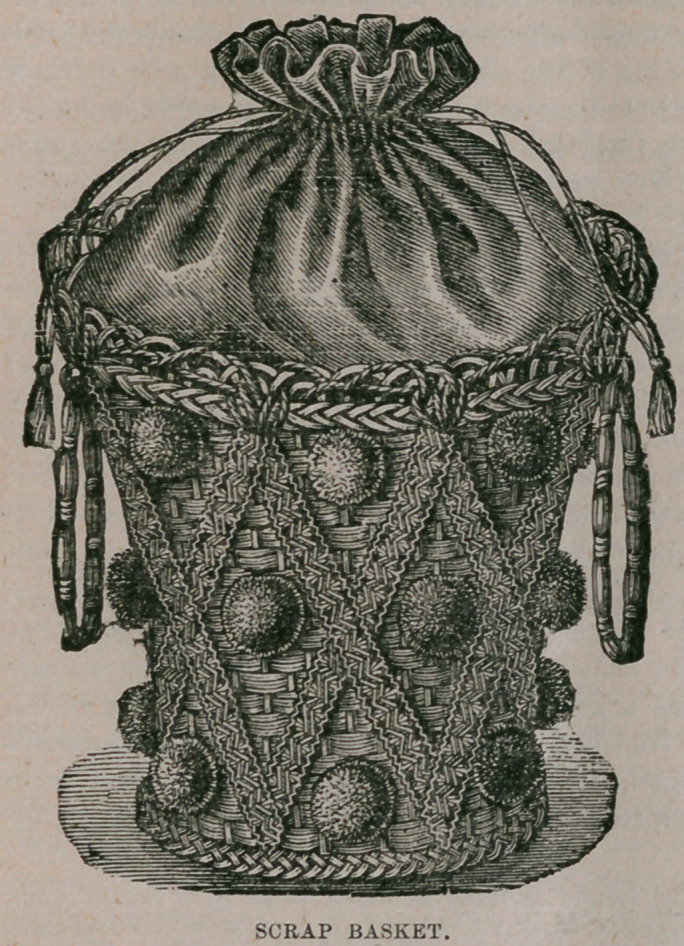# Household

**Published:** 1889-01

**Authors:** 


### HOUSEHOLD.

Scrap Basket.—The scrap basket has become as much an article of furnishing for library and sitting room as is the table itself. So elaborate and elegant are many of them, that they seem almost too good to use, and while we may admire at a distance these works of art, we prefer for use something which will not look shabby too soon, and in its decayed elegance be more forlorn than a less expensive and more durable basket in the beginning. Our illustration shows a handsome basket, substantially fitted up, and likely to stand pretty good service. As may be seen, it is a circular flaring basket of fancy straw (gilded) with bands of red and olive, sewed on crosswise to form a diamond pattern, and having balls of olive wool between the bands of felt. The basket is lined inside with red cashmere, allowing the material to come above and form the bag, which is drawn up with cord and tassels of olive wool. The bands of felt are pinked on the edges and worked in the center with vandyked lines of olive silk and crewels. The olive bands are worked in chain stitch and point russe, with two shades of pink, and the red bands with two shades of bronze. The bands are then sewed on to the basket, and the balls added. If a fuller trimming is desired, a double box plaiting of the felt, pinked on both edges and plaited through the middle is put around the top and bottom of the basket.

SCRAP BASKET.

Frozen Coffee.—Take two quarts of fresh filtered, or spring water, if obtainable, bring it to the boil, then add half a pound of the best old government Java coffee, roasted and ground, stir well together, cover and set aside on the range to infuse. Stir occasionally for the first ten minutes, than let it stand in a warm place till well settled. Now strain the coffee clear, through a fine muslin cloth and add water to make two quarts, dissolve one pound of pulverized sugar in it, and set aside to cool; then pour it into the freezer, add the whites of two eggs, and freeze the mixture to a softish texture. This frappé is generally served in high glasses. On the continent of Europe this ice is called "café mousseux," also "café frappé a la glacé." The fourth part of a vanilla bean is also sometimes infused in the coffee when making it, and tends to heighten the aroma of the coffee. Some persons also add half a pint of rich cream to it before freezing. These additions, however, are matters of taste.

The Coffee-Pot.—It seems a very simple thing to make good coffee, and yet in no one thing do as many housekeepers fail ; the coffee-pot, however, is largely responsible for the failure. Many housekeepers who have servants think the cook will not fail to attend to the cleaning of the coffee pot, while others who do their own work attach no importance to the matter, and allow the grounds and cold coffee to stand in it for several days without emptying, and at the same time wonder why they never have good coffee, notwithstanding the best article is bought, well parched and carefully made.

To have the beverage perfect, it is necessary that great attention be given the coffee pot. It should be emptied after each meal, well washed inside and outside in clean, hot soap-suds, then rinsed in boiling water first and then in cold water, after which it should be well dried and set in the sun. Occasionally (once a week) it is well to put a tablespoonful of soda in the coffee-pot, fill with boiling water and set on the stove. This cleanses and purifies it. This attention to what may seem a very small matter is of the utmost importance to the housekeeper, and will be found well worth the slight extra trouble by all who try it. After a good article of coffee, nothing, then, is so important, in order to have good coffee, as a clean coffee-pot.

Danger in Lead Pipes.—It is claimed to be proven beyond all doubt that waters which circulate or stand in leaden pipes or vessels, not only take up particles of lead through mechanical action due to friction, but attack the metal, the result of this being generally lead carbonate. According to the most eminent authorities in this line, minute quantities of lead thus introduced into and accumulating in the system, must rank among the causes of anæmia and defective nutrition in large towns.

To Clear Waste Pipes.—"Just before retiring at night pour into the clogged pipe enough liquid soda lye to fill the 'trap' or bent part of the pipe. Be sure that no water runs in it until the next morning. During the night the lye will convert all the offal into soft soap, and the first current of water in the morning will wash it away and clear the pipe clean as new."

To Clean Ivory Ornaments.—Ivory ornaments are quickly cleaned by brushing them with a new, not very sharp toothbrush, to which little soap is given ; then rinse the ornament in lukewarm water. Next dry the trinket and brush a little, and continue brushing until the luster reappears, which can be increased by pouring some alcohol upon the brush and applying it to the trinket. Should this have become yellow, dry it in a gentle heat and it will appear as if new.

To Wash Plush Cloaks.—First hang your cloak on the line and get all the dust out of it with a switch. Then spread it on the back of a chair and sponge every inch of it with warm rain water and a little ammonia. Take a dry sponge and rub the cloak until it is almost dry. Rub both ways, back and forth, until the nap is thoroughly raised. Lastly hang the cloak in the sun until it is perfectly dry and brush it with a soft brush. The result will surprise you for the plush will look like new. Don't be afraid to try it ; it has been tested.

## Figures and Tables

**Figure f1:**